# Regular treatment with formoterol and an inhaled corticosteroid versus regular treatment with salmeterol and an inhaled corticosteroid for chronic asthma: serious adverse events

**DOI:** 10.1590/S1516-31802010000500014

**Published:** 2010-09-02

## Abstract

**BACKGROUND:**

An increase in serious adverse events with both regular formoterol and regular salmeterol in chronic asthma has been demonstrated in comparison with placebo in previous Cochrane reviews. This increase was significant in trials that did not randomise participants to an inhaled corticosteroid, but less certain in the smaller numbers of participants in trials that included an inhaled corticosteroid in the randomised treatment regimen.

**OBJECTIVES:**

We set out to compare the risks of mortality and non-fatal serious adverse events in trials which have randomised patients with chronic asthma to regular formoterol versus regular salmeterol, when each are used with an inhaled corticosteroid as part of the randomised treatment

**SEARCH STRATEGY:**

Trials were identified using the Cochrane Airways Group Specialised Register of trials. Manufacturers’ web sites of clinical trial registers were checked for unpublished trial data and Food and Drug Administration (FDA) submissions in relation to formoterol and salmeterol were also checked. The date of the most recent search was July 2009.

**SELECTION CRITERIA:**

Controlled clinical trials with a parallel design, recruiting patients of any age and severity of asthma were included if they randomised patients to treatment with regular formoterol versus regular salmeterol (each with a randomised inhaled corticosteroid), and were of at least 12 weeks duration.

**DATA COLLECTION AND ANALYSIS:**

Two authors independently selected trials for inclusion in the review and extracted outcome data. Unpublished data on mortality and serious adverse events were sought from the sponsors and authors.

**MAIN RESULTS:**

Eight studies met the eligibility criteria of the review recruiting 6,163 adults and adolescents. There were seven studies (involving 5,935 adults and adolescents) comparing formoterol and budesonide to salmeterol and fluticasone. All but one study administered the products as a combined inhaler, and most used formoterol 50 mcg and budesonide 400 mcg twice daily versus salmeterol 50 mcg and fluticasone 250 mcg twice daily. There were two deaths overall (one on each combination) and neither were thought to be related to asthma. There was no significant difference between treatment groups for non-fatal serious adverse events, either all-cause (Peto OR 1.14; 95% CI 0.82 to 1.59, I2 = 26%) or asthma-related (Peto OR 0.69; 95% CI 0.37 to 1.26, I2 = 33%). Over 23 weeks the rates for all-cause serious adverse events were 2.6% on formoterol and budesonide and 2.3% on salmeterol and fluticasone, and for asthma-related serious adverse events, 0.6% and 0.8% respectively. There was one study (228 adults) comparing formoterol and beclomethasone to salmeterol and fluticasone, but there were no deaths or hospital admissions. No studies were found in children.

**AUTHORS’ CONCLUSIONS:**

The seven identified studies in adults did not show any significant difference in safety between formoterol and budesonide in comparison with salmeterol and fluticasone. Asthma-related serious adverse events were rare, and there were no reported asthma-related deaths. There was a single small study comparing formoterol and beclomethasone to salmeterol and fluticasone in adults, but no serious adverse events occurred in this study. No studies were found in children. Overall there is insufficient evidence to decide whether regular formoterol and budesonide or beclomethasone have equivalent or different safety profiles from salmeterol and fluticasone.

Attending Physician, Discipline of Pneumonology Universidade Federal de São Paulo (Unifesp), São Paulo, Brazil.

**This review should be cited as**:

Cates CJ, Lasserson TJ. Regular treatment with formoterol and an inhaled corticosteroid versus regular treatment with salmeterol and an inhaled corticosteroid for chronic asthma: serious adverse events. Cochrane Database of Systematic Reviews 2010, Issue 1. Art. No.: CD007694. DOI: 10.1002/14651858.CD007694.pub2.

**FURTHER INFORMATION:**

Centro Cochrane do Brasil Rua Pedro de Toledo, 598 Vila Clementino — São Paulo (SP) — Brasil CEP 04039-001 Tel. (+55 11) 5579-0469/5575-2970 http://www.centrocohranedobrasil.org.br/

**This section was edited under the responsibility of the Brazilian Cochrane Center**

The full review is available (free access) from: http://www.cochranejournalclub.com/formoterol-vs-salmetrol-adverse-events-clinical/pdf/CD007694_full.pdf

## Comments

Inhalatory medications (inhalatory medications and bronchodilators) are the basis for treatment of the stable phase of asthma and for chronic obstructive pulmonary disease (COPD). The pathogenesis of asthma results from an inflammatory process in the airways that leads to contraction of the smooth musculature and triggers the symptoms of coughing, expectoration and dyspnea. Therefore, the treatment for stable asthma is based on the use of inhalatory corticoids for all patients with persistent conditions (mild, moderate or severe). For some patients, this is used in association with long-duration bronchodilators (LABAs) because of the clinical and functional state (spirometry). Inhalatory corticoids include beclomethasone, budesonide, fluticasone and ciclesonide, while formoterol and salmeterol are LABAs. Through previous systematic reviews, it has been demonstrated that use of LABAs alone may lead to higher mortality among asthma patients.

The aim of this systematic review was to compare occurrences of mortality and non-fatal severe adverse effects in clinical trials that randomized patients with asthma to make regular use of formoterol versus regular use of salmeterol, used in association with inhalatory corticoid as part of the randomized treatment. Eight studies were assessed in the systematic review, totaling 6,163 patients studied. The associations studied were formoterol + budesonide, salmeterol + fluticasone and formoterol + beclomethasone.

There were two deaths in the studies, but none related to asthma. There was no significant difference between the treatment groups regarding occurrences of non-fatal severe adverse effects, either for all causes (2.6% in the formoterol group and 2.3% in the salmeterol group) or for asthma-related causes (0.6% in the formoterol group and 0.8% in the salmeterol group). No studies on children were found. Only one study evaluating the association between formoterol and beclomethasone was found, but there were no severe adverse effects or deaths in this study.

The studies evaluated in this systematic review did not show any significant difference regarding safety between the associations of formoterol + budesonide and salmeterol + fluticasone. Severe adverse events were rare and there were no asthma-related deaths.

Therefore, through this systematic review, it can be concluded that the use of inhalatory corticosteroid with LABA is safe and that it constitutes an excellent therapeutic alternative for patients with persistent asthma.

